# Chemical Bonding and σ-Aromaticity in Charged Molecular Alloys: [Pd_2_As_14_]^4−^ and [Au_2_Sb_14_]^4−^ Clusters

**DOI:** 10.1038/s41598-017-00867-5

**Published:** 2017-04-11

**Authors:** Xue-Rui You, Lin-Yan Feng, Rui Li, Hua-Jin Zhai

**Affiliations:** 1 Nanocluster Laboratory, Institute of Molecular Science, Shanxi University, Taiyuan, 030006 China; 2 State Key Laboratory of Quantum Optics and Quantum Optics Devices, Shanxi University, Taiyuan, 030006 China

## Abstract

We report a computational study on the structures and bonding of a charged molecular alloy *D*_2*h*_ [Pd_2_As_14_]^4−^ (**1**), as well as a model *D*_2*h*_ [Au_2_Sb_14_]^4−^ (**2**) cluster. Our effort makes use of an array of quantum chemistry tools: canonical molecular orbital analysis, adaptive natural density partitioning, natural bond orbital analysis, orbital composition analysis, and nucleus independent chemical shift calculations. Both clusters consist of two X_7_ (X = As, Sb) cages, which are interconnected via a M_2_ (M = Pd, Au) dumbbell, featuring two distorted square-planar MX_4_ units. Excluding the Pd/As or Au/Sb lone-pairs, clusters **1** and **2** are 50- and 44-electron systems, respectively, of which 32 electrons are for two-center two-electron (2c-2e) As-As or Sb-Sb σ bonds and an additional 16 electrons in **1** for 2c-2e Pd-As σ bonds. No covalent Pd-Pd or Au-Au bond is present in the systems. Cluster **1** is shown to possess two globally delocalized σ electrons, whereas **2** has two σ sextets (each associated with an AuSb_4_ fragment). Thus, **1** and **2** conform to the (4*n* + 2) Hückel rule, for *n* = 0 and 1, respectively, rendering them σ-aromaticity.

## Introduction

The advances in cluster science have led to a large body of intermetallic and multinary clusters, which are blurring the lines between nanomaterials and cluster chemistry^1^. Structural analyses of large metallic clusters^2–4^ showed that their cores are discrete pieces of bulk solids, being stabilized by organometallic ligands on the periphery. For binary transition-metal clusters^5–7^, solid-state alloy phases were observed as the structural cores; yet unique and “molecular” structures were also discovered. Transition from molecular to solid-state structures seems to depend upon the elements involved, as well as the supporting ligands in the cluster^5, 8^. Transition metal/main group binary anions, in particular those involving the group 15 elements (that is, the so-called Zintl anions), are an expanding field of synthetic chemistry^1, 9–11^.

In alloy-like clusters [Ni_5_Sb_17_]^4–^ ^12^, [Zn_9_Bi_11_]^5−^ ^13^, and ternary [Pd_3_Sn_8_Bi_6_]^4−^ ^14^, most of transition metal atoms are situated on the surface, offering unusual geometric shapes and complicated electronic structure. Transition metals may also serve as oxidation agents: Pb_10_^2−^ was obtained through Pb_9_^4−^ as oxidized by an Au(I) precursor^15^. Anions with low nuclearities such as Sb_*n*_^*n*−^ (*n* = 3, 5) and Bi_*n*_^3−^ (*n* = 2, 3) were isolated or stabilized by transition metals, suggesting the existence of [E_*n*_]^*n*−^ in solutions^16–18^. For higher nuclearities, Bi_*n*_^3−^ (*n = *7, 11) were obtained in the presence of Cr and Ga, respectively^19, 20^.

Main group elemental cages as “naked” cluster anions, in particular heptapnictide trianions [E_7_]^3−^ (E = P, As, Sb)^21^, were actively explored in transition metal binary anions. Dissolution of A_3_E_7_ alloys (A = alkali metal; E = P, As, Sb) in polar, nonprotic solvents (ethylenediamine or liquid ammonia) are known to yield solutions of [E_7_]^3−^ cages. The cages have nortricylane-like structures, in which the E-E interactions are two-center two-electron (2c-2e) bonds. An extensive series of synthetic clusters with intact [E_7_]^3−^ (E = P, As) anions were reported: [(As_7_)Sn(As_7_)]^4−^ ^22^, [(As_7_)Au_2_(As_7_)]^4−^ ^23^, [(As_7_)Pd_2_(As_7_)]^4−^ ^24, 25^, [(P_7_)Cu_2_(P_7_)]^4−^ ^26^, [(P_7_)Zn(P_7_)]^4−^ ^26^, and [(P_7_)Cd(P_7_)]^4−^ ^26^. In these clusters, [E_7_]^3−^ anions were believed to coordinate to either a M^2+^ center, or a Cu_2_^2+^, Au_2_^2+^, and Pd_2_^6+^ dumbbell, whose exact nature of bonding was seldom elucidated in full detail in the literature. Negative charges in the cluster anions were concluded to be localized on the two-fold coordinate E atoms and the central transition metal sites.

In the past years, we have been interested in exploring the structural, electronic, and bonding properties of gas-phase clusters^23–27^ and synthetic cluster compounds^36–38^. In the present contribution, we shall report on a quantum chemical study on the structure and bonding of a “charged molecular alloy” cluster: *D*_2*h*_ [Pd_2_As_14_]^4−^ (**1**). This cluster was crystallized in the form of [K([2.2.2]crypt)]_4_[Pd_2_As_14_]·5en (en = ethylenediamine)^24^, by Eichhorn and coworkers in 2002, which consists of two [As_7_] cages interconnected via a Pd_2_ dumbbell. The Pd centers are coordinated by As in a distorted square-planar fashion, featuring two PdAs_4_ fragments. In this bulk compound, the [Pd_2_As_14_]^4−^ tetraanion is effectively stabilized by four K^+^ counter-ions. Eichhorn and coworkers proposed the [As_7_]^5−^ and Pd_2_^6+^ building blocks, the formal Pd(III) centers, and an axial Pd-Pd bond in **1**. However, there has been no quantum chemical study on this system so far, to the best of our knowledge, and the nature of bonding in the cluster remains elusive.

Herein we have performed a detailed computational study on the [Pd_2_As_14_]^4−^ (**1**) cluster at the PBE0 level^39^ of density-functional theory (DFT)^40^ and elucidated its nature of chemical bonding using a range of state-of-the-art quantum chemistry tools: canonical molecular orbital (CMO) analysis, adaptive natural density partitioning (AdNDP)^41^, natural bond orbital (NBO)^42^ analysis, orbital composition analysis, and nucleus independent chemical shift (NICS)^43^. To further ensure the computational reliability for a naked multiply charged anion, conductor-like polarizable continuum mode (C-PCM)^44–47^ calculation as an alternative method has also been carried out for **1** to take into account the solvation effects, whose results many people believe should be closer to the truth for a synthetic cluster compound in the bulk: a multiply charged anion being stabilized by bulky ligands including counter-ions^24^. The computational data allows an in-depth understanding of the nature of bonding in **1**, which turns out to possess two delocalized σ electrons within the square-planar PdAs_4_ fragments, rendering σ-aromaticity for the charged molecular alloy according to the (4*n* + 2) Hückel rule, for *n* = 0. Based on the findings in [Pd_2_As_14_]^4−^ (**1**), we have further explored the rational design of a model charged molecular alloy, *D*_2*h*_ [Au_2_Sb_14_]^4−^ (**2**). Cluster **2** has two more valence electrons than **1** and yet differs markedly from the latter in terms of chemical bonding. **2** has two square-planar AuSb_4_ fragments, each supporting 6σ delocalized electrons (that is, σ sextet), which conform to the (4*n* + 2) Hückel rule for aromaticity, for *n* = 1. Clusters **1** and **2** provide new examples for σ-aromaticity, from gas-phase clusters to synthetic solid-phase compounds. It is stressed that **1** and **2** are relatively large cluster systems and bonding analyses are rather challenging; the current level of understanding for **1** and **2** is largely attributed to the powerful AdNDP tool^41^ for chemical bonding analyses, which was not possible even a couple of years ago. The concept of σ-aromaticity, in particular σ sextets, was discussed recently in a synthetic [Au_2_Sb_16_]^4−^ compound^38^.

## Results and Discussion

### Cluster Structure of [Pd_2_As_14_]^4−^

**Figure 1:**
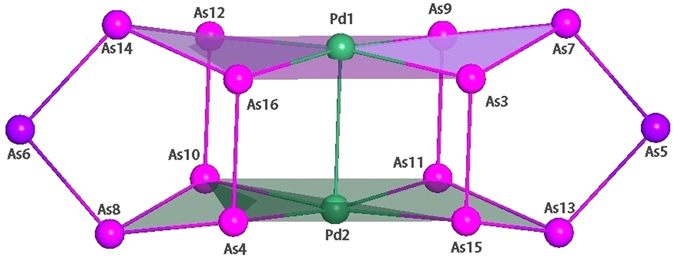
Optimized *D*_2*h*_ structure of [Pd_2_As_14_]^4−^ (**1**) cluster at the PBE0/def2-TZVP level. Bond distances (in Å): As-As, 2.38–2.46; Pd-Pd, 2.75; As-Pd, 2.50. Bond angles (in degrees): ∠AsPdAs, 76.30–172.68; ∠PdAsAs, 93.66–100.25; ∠AsAsAs, 78.00–108.42.

We obtained the initial coordinates from crystal data of the Eichhorn paper^24^ and fully reoptimized the structure of [Pd_2_As_14_]^4−^ at the PBE0/def2-TZVP level. The ultimate cluster structure is *D*_2*h*_ [Pd_2_As_14_]^4−^ (**1**). Calculated bond distances and bond angles are summarized in Fig. 1 and Table 1. The Cartesian coordinates for **1** are presented in Table S1 in the Supplementary Information, along with those of *D*_2*h*_ [Au_2_Sb_14_]^4−^ (**2**). Overall, the calculated bond distances of **1** are highly coherent with the experimental measurements (Table 1).

**Table 1:** Optimized geometric structure of *D*_2*h*_ [Pd_2_As_14_]^4−^ (**1**) cluster at the PBE0/def2-TZVP level.

bond^a^		Pd(1)-Pd(2)	Pd(1/2)-As(iii)	As(i)-As(ii)	As(ii)-As(iii)	As(iii)-As(iii)^b^
bond distance	calc.	2.75	2.50	2.38	2.46	2.43
	exptl.^c^	2.7144(6)	2.4770(5)	2.3616–2.4455(7)		
Wiberg index		0.250	0.543	1.089	0.903	1.030
Pd/As center		Pd		As(i)	As(ii)	As(iii)
natural charge^d^		−0.671		−0.602	−0.161	−0.102

The calculated bond distances (in Å) are compared with those from synthetic experiment. Also presented are the Wiberg bond indices and natural atomic charges (in |e|) via the natural bond orbital (NBO) analysis.

^a^The As atoms in [Pd_2_As_14_]^4−^ (**1**) may be classified into three types. As(i): two bridging As atoms; As(ii): four As atoms that are tricoordinated with As; As(iii): eight As atoms linked to the Pd atoms.

^b^Only four As(iii)-As(iii) bonds are present in **1**, which link the two square-planar PdAs_4_ units (Fig. 1).

^c^Experimental data from ref. 24.

^d^Charge per Pd or As atom.

The cluster can be viewed as two As_7_ cages interconnected by a Pd_2_ dimer (Fig. 1), as described by Eichhorn and coworkers^24^. The Pd centers hold the As_7_ cages together via two distorted square-planar PdAs_4_ units. The fourteen As atoms in **1** may be classified into three subgroups, that is, *As(i)*: two bridging As atoms at the far left and far right of the cluster; *As(ii)*: four As atoms that are tricoordinated with As; *As(iii)*: eight As atoms linked to Pd centers. For the As-As interactions, the four As(ii) centers are tricoordinated with two As(i) and eight As(iii), forming 12 As-As links. These As-As distances range from 2.38 to 2.46 Å (Table 1). The two PdAs_4_ units are connected via four interlayer As(iii)-As(iii) links, whose distances are 2.43 Å. In total, there are 16 As-As links in **1**. According to the recommended covalent radii by Pyykkö^48^, typical As-As single and As=As double bonds are around 2.42 and 2.28 Å, respectively. Thus, all 16 As-As links are single bonds.

In terms of the Pd-As interactions in square-planar PdAs_4_ units, the eight Pd-As links have distances of 2.50 Å, which are to be compared to the recommended distances of Pd-As single (2.41 Å) and Pd=As double (2.31 Å) bonds^48^. Therefore, the Pd-As bonds in **1** are weaker than single bonds; we tentatively assign them as single bonds. The peripheral As(iii)-As(iii) links within two PdAs_4_ planes are markedly elongated (As12–As16: 3.09 Å; As16-As3: 3.93 Å) with respect to the As-As bonds discussed above, indicating that no direct As-As interactions are present in the periphery.

The calculated Pd-Pd distance is 2.75 Å, which turns out to be markedly longer than a single bond (2.40 Å)^48^. Even larger Pd-Pd distance was reported in the literature: [Pd_2_@Ge_18_]^4−^ (2.831 Å)^49^. In the Eichhorn paper^24^, an axial Pd-Pd bond in **1** was explicitly claimed. We believe the Pd-Pd covalent bonding in **1** is relatively minor (see below), and the Pd atoms can be viewed as isolated, single atoms. Note that despite the fact that the Pd centers in **1** are not in Pd(0) configuration, a “dispersion interaction” mechanism, similar to aurophilicity in Au-Au clusters^50, 51^, should help stabilize the Pd_2_ dumbbell in **1**. Arguably, the open-shell configuration of Pd centers in **1** can facilitate stronger Pd-Pd dispersion interaction as compared to Pd d^10^ centers. The calculated Wiberg index for Pd-Pd amounts to 0.250 (Table 1), which is moderate in spite of the absence of conventional Pd-Pd bond in the system. Bader analysis yields a similar Pd-Pd bond index of 0.170.

Multiply charged cluster anions are not uncommon as synthetic compounds, as well as in computational modeling. Tetraanion *D*_2*h*_ [Pd_2_As_14_]^4−^ (**1**) is intrinsically unstable due to intramolecular Coulomb repulsion. Our outer valence green's function (OVGF) calculations for **1** give a negative electron binding energy of −5.37 eV as anticipated, which is routine for a highly charged tetraanion. Nonetheless, we have analyzed the wavefunction stability for tetraanion **1** and the result indicates indeed that the wavefunction is stable under the perturbations considered.

In response to the concern of one referee, we further technically designed a model neutral cluster, *C*_*i*_ [Pd_2_As_14_K_4_] (**3**), in which [Pd_2_As_14_]^4−^ (**1**) tetraanion is electrostatically stabilized by four K^+^ cations. Geometry optimizations and frequency calculations show that the tetraanion **1** and the model neutral cluster **3** (see Fig. S1 in the Supplementary Information) have remarkably similar structures with respect to that in the synthetic bulk compound (Fig. S2). The structural and electronic integrity of **1** is fully maintained in **3**. The structure of **3** is closely relevant to and yet significantly simpler than the [K([2.2.2]crypt)]_4_[Pd_2_As_14_]·5en bulk compound^24^, rendering the former cluster a valuable neutral model of the latter compound. Alternatively, the referee’s concern of the computational reliability for a bare tetraanion such as **1** can be addressed using the C-PCM calculations^44–47^, in which the solvation effects (with dielectric constant ε_*r*_ (ethylendiamine) = 12.9) are considered. The optimized structure of **1** in the C-PCM calculations is presented in Fig. S3.

Overall, we have now four sets of structural data for [Pd_2_As_14_]^4−^: (*a*) optimized PBE0 structure of [Pd_2_As_14_]^4−^ (**1**) tetraanion (Fig. S1a); (*b*) optimized [Pd_2_As_14_K_4_] (**3**) as a model neutral salt complex at PBE0 (Fig. S1b); (*c*) optimized tetraanion [Pd_2_As_14_]^4−^ (**1**) with the inclusion of solvation effects (Fig. S3); (*d*) synthetic bulk crystal structure [K([2.2.2]crypt)]_4_[Pd_2_As_14_]·5en from ref. 24 (see Fig. S2). A close comparison indicates that the computational data for bare tetraanion in set (a), which some people consider to be questionable or unreliable, are actually a faithful reproduction of the crystal data^24^; the computational bond distances of As-As and As-Pd are typically 0.01–0.03 Å longer than the experimental data, due to Coulomb repulsion. This observation suggests that tetraanion is not a problem for PBE0, at least in terms of structural optimization. The optimized structures in sets (b) and (c) are even closer to the experiment, with typical errors of 0.00–0.02 Å. However, the improvement is very limited, largely because the computational data of set (a), that is, bare tetraanion at PBE0 without the solvation effects, appear to be excellent. In all cases, the optimized As-As distances deviate very slightly from each other and can all be assigned as single bonds.

All valence electrons are bound in **3** (Fig. S1b) and the OVGF calculations give a positive ionization potential of 5.43 eV, which is in contrast to the above-mentioned negative value for **1** (Fig. S1a). This difference between **3** and **1** is due to electrostatic stabilization in **3**, between four K^+^ cations and tetraanion **1**, as pointed out above. Alternatively, with C-PCM calculations (Fig. S3) to account for solvation effects, all occupied CMOs (that is, the Kohn-Sham orbitals) of **1** become highly negative in energy eigenvalues. Figure S4 depicts the highest occupied and lowest unoccupied molecular orbitals (HOMO and LUMO) of **1** (with solvation effects) and **3**, as well as additional low-lying CMOs down to HOMO-4. These frontier CMOs are the same except that their energy order changes slightly due to the perturbation of K^+^ cations in **3**, as anticipated. Furthermore, we note explicitly that the calculated CMOs of **1** with and without solvation effects are identical with each other in shapes and in energy order, except that those with solvation effects are shifted to deeper energies.

The above comparative data between **1** (without or with solvation effects) and **3** suggest that the present results for *D*_2*h*_ [Pd_2_As_14_]^4−^ (**1**) should generally be considered reliable for the state-of-the-art quantum chemistry. The four K^+^ counter-ions in **3** are associated with the Pd centers and two As(i) bridging atoms, which carry the majority of four extra electrons in **1** (see below for details). Indeed, the K^+^ counter-ions in the bulk compound occupy similar positions with respect to Pd and As(i) atoms, with slight deviations for two out of eight K^+^ ions (Fig. S2) due to steric effects of the bulky ligands. Therefore, we conclude that cluster **3** (Fig. S1b) is a good and simplified neutral model for the bulk compound, whereas bare tetraanion **1** (Fig. S1a) and tetraanion **1** with solvation effects (Fig. S3) are also reliable computational structures. All four sets of computational and experimental structures actually represent the same chemical entity.

### Chemical Bonding in *D*_2*h*_ [Pd_2_As_14_]^4−^: Pd-Pd Bonding and σ-Aromaticity

**Figure 2:**
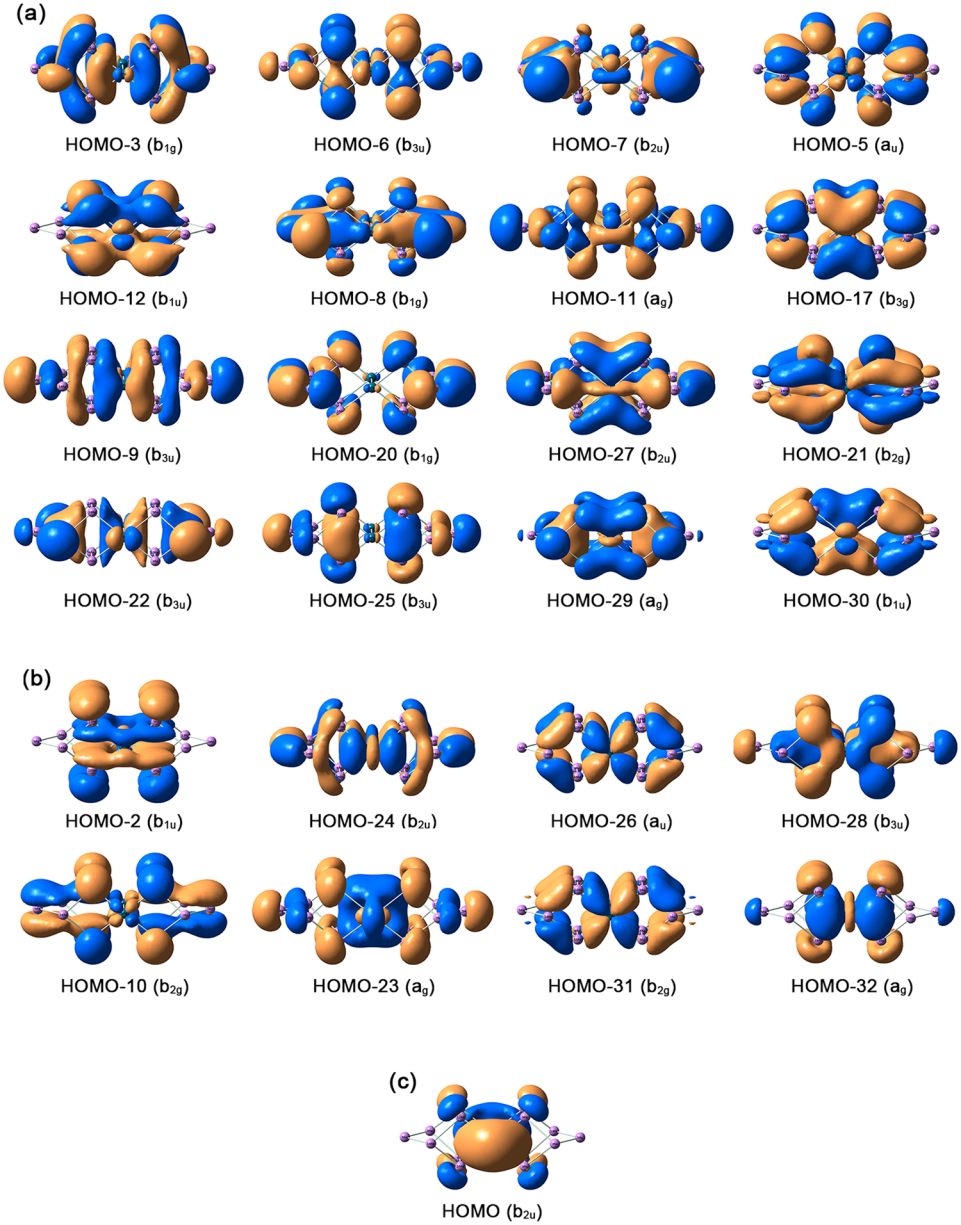
Pictures of selected canonical molecular orbitals (CMOs) in [Pd_2_As_14_]^4−^ (**1**), calculated at the PBE0/def2-TZVP level. (**a**) CMOs for 16 two-center two-electron (2c-2e) As-As σ bonds. (**b**) CMOs for eight 2c-2e Pd-As σ bonds. (**c**) CMO for the globally delocalized σ bond. Additional CMOs are 6 pairs of nonbonding Pd 4d electrons (including four lone-pairs), fourteen As 4s lone-pairs, and two As 4p lone-pairs; see Fig. S5 in the Supplementary Information.

Considering the Pd 4d^10^ and As 4s^2^4p^3^ configurations, *D*_2*h*_ [Pd_2_As_14_]^4−^ (**1**) possesses 94 valence electrons in total (including 4 extra charges). It is a relatively large “charged molecular alloy” cluster, and thus an in-depth chemical bonding analysis is difficult. However, the structural description on the basis of bond distances, as outlined in the previous section, allows simplification of the task, because there exist clearly 16 As-As and 8 Pd-As single bonds in **1**, which consume 48 electrons. Furthermore, according to our recent work on [Sb_3_Au_3_Sb_3_]^3−^ all-metal sandwich^36, 37^, the As 4s^2^ electrons in **1** are anticipated to well behave as lone-pairs (14 of them, consuming 28 electrons). The above-mentioned single bonds and lone-pairs use 76 electrons out of 94 in **1**, whose corresponding CMOs are depicted in Fig. 2a and b, as well as in Fig. S5b.

Technically, the 14 CMOs for As 4s^2^ lone-pairs (Fig. S5b) consist of 7 pairs of constructive/destructive combination between the two As_7_ cages (left versus right), which are readily transformed to two sets of 7 orbitals, each for one As_7_ cage. For example, HOMO-46 and HOMO-45 are responsible for one completely bonding 7c-2e σ orbital on the left As_7_ cage and one on the right. Of these 7 orbitals in the As_7_ cage, one is for the lone-pair on As(i), and the remaining 6 are again three pairs of constructive/destructive combination between two As_3_ triangles (front versus rear). The three orbitals for each As_3_ are in a typical bonding/nonbonding/antibonding combination, eventually leading to three As 4s^2^ lone-pairs. For As-As and Pd-As single bonds, we only take the Pd-As bonds as an example (Fig. 2b). Here, HOMO-2, HOMO-10, HOMO-28, and HOMO-32 involve substantial As components, whereas the remaining four CMOs have significant Pd 4d components. Thus, it can be roughly considered that two Pd centers contribute 8 electrons for Pd-As bonding, with the eight As(iii) centers matching the remaining half, which is an ideal case for eight Pd-As single bonds within two PdAs_4_ units.

**Figure 3:**
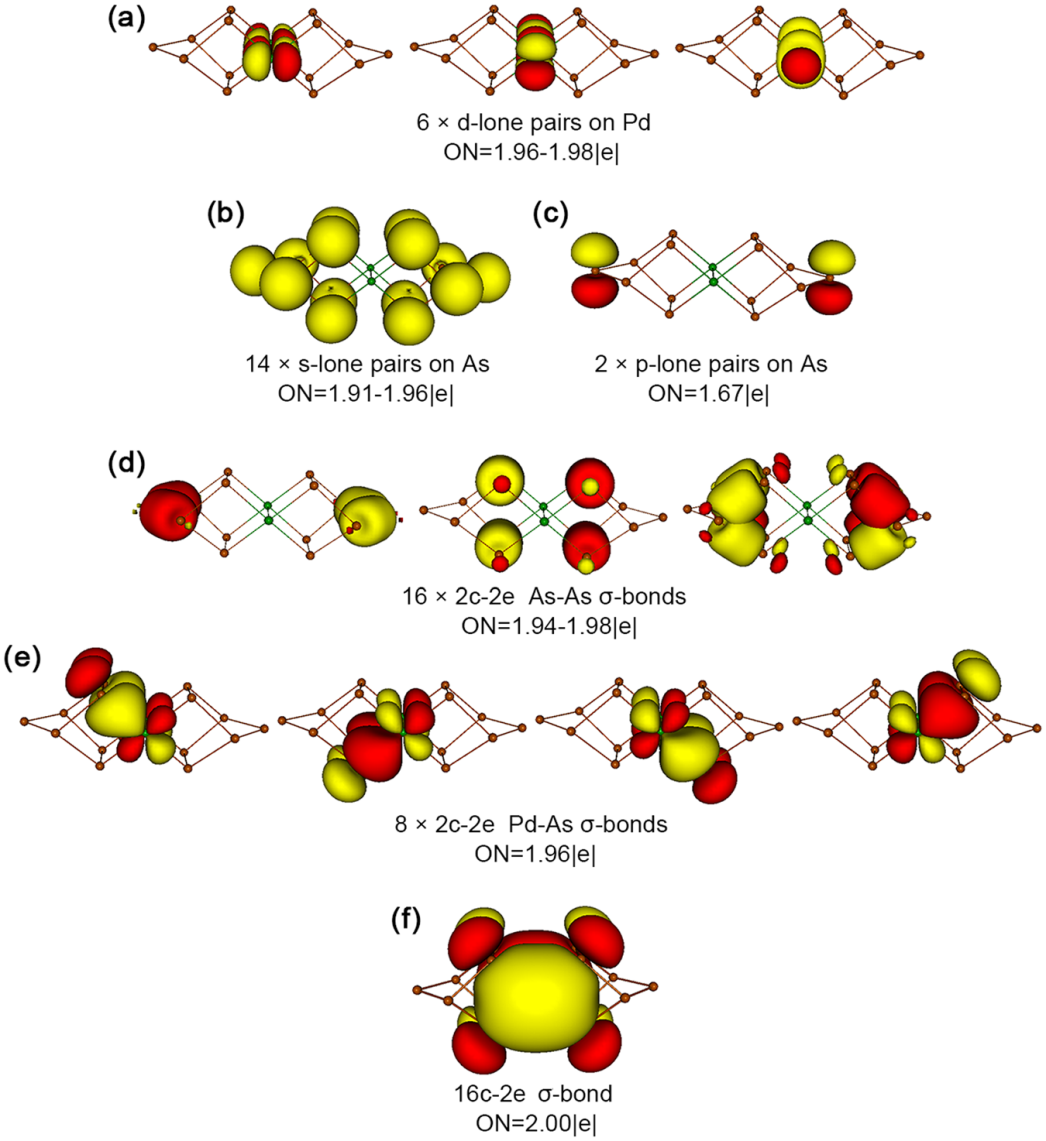
Bonding pattern for *D*_2*h*_ [Pd_2_As_14_]^4−^ (**1**) cluster as revealed from the adaptive natural density partitioning (AdNDP) analysis. Occupation numbers (ONs) are shown.

While the above CMO analyses can be difficult to comprehend, the AdNDP method developed by Boldyrev and coworkers^41^ offers an alternative, straightforward way to describe such bonding elements. AdNDP is an extension of NBO analysis. It represents the electronic structure of a molecular system in terms of *n*-center two-electron (*n*c-2e) bonds, with the value of *n* ranging from one to the total number of atoms in the molecule. Therefore, AdNDP analysis recovers not only the classical Lewis elements (lone-pairs and 2c-2e bonds), but also delocalized *n*c-2e bonds. Indeed, the AdNDP data for cluster **1** elegantly recover fourteen As 4s^2^ lone-pairs (Fig. 3b), sixteen 2c-2e As-As σ bonds (Fig. 3d), and eight 2c-2e Pd-As σ bonds (Fig. 3e).

Of the remaining 18 electrons in **1**, the 4 extra charges are situated on two As(i) and two Pd centers (Table 1), which carry a charge of −0.602 and −0.671 |e|, respectively. To be precise, the charge on an As(i) center smears moderately along the As8-As6-As14 or As7-As5-As13 chains (Fig. 1), which collectively carry a charge of −0.924 |e|, nearly ideal for a single charge. Likewise, the charge on a Pd center smears moderately over a PdAs_4_ unit, collectively carrying a charge of −1.079 |e|. From Fig. S5a, one can identify 6 CMOs that are largely Pd 4d based, which are either lone-pairs or nonbonding. Specifically, HOMO-19/HOMO-13 are primarily the constructive/destructive combination of Pd d_yz_ atomic orbitals (AOs) between two Pd centers; these can be transformed to two Pd d_yz_ lone-pairs. Likewise, HOMO-18/HOMO-14 are readily transformed to two Pd ${{\rm{d}}}_{{{\rm{z}}}^{2}}$ lone-pairs. On the other hand, HOMO-15 and HOMO-16 are constructive/destructive combination of Pd ${{\rm{d}}}_{{{\rm{x}}}^{2}-{{\rm{y}}}^{2}}$ or d_xz_ AOs; these are nonbonding in nature between the Pd centers but cannot be transformed to Pd 4d lone-pairs (because their corresponding destructive/constructive CMOs are used for Pd-As σ bonds). In short, these CMOs correspond to four Pd 4d lone-pairs and two pairs of nonbonding Pd 4d electrons. The above 6 CMOs are presented as six Pd 4d lone-pairs in the AdNDP analysis (Fig. 3a); the latter is an effective and approximate description only.

The “missing” complementary Pd 4d based orbitals (four of them) are involved in the Pd-As bonding (Fig. 2b). Furthermore, two As 4p lone-pairs on As(i) are identified in the CMOs (Fig. S5c) and AdNDP data (Fig. 3c). In short, these lone-pairs or nonbonding CMOs further consume 16 electrons. As a consequence, 92 electrons in **1** (out of 94 in total) can be faithfully described as classical Lewis elements: Pd 4d, As 4s, and As 4p lone-pairs (or nonbonding CMOs); 2c-2e As-As single bonds; and 2c-2e Pd-As single bonds.

Interestingly and remarkably, the only delocalized bond in **1** is the HOMO (Fig. 2c), which is a σ bond situated on the two square-planar PdAs_4_ layers. Note that the delocalized σ bond, HOMO, is also reproduced in AdNDP (Fig. 3f). At this point, we shall summarize our effort in CMO analyses described above. In brief, all 47 valence CMOs (94 valence electrons) are presented in Fig. S5 and Fig. 2. These CMOs are categorized into six subsets: (1) six Pd 4d lone-pairs or nonbonding electrons (Fig. S5a), (2) fourteen As 4s lone-pairs (Fig. S5b), (3) two As 4p lone-pairs for bridging As(i) sites (Fig. S5c), (4) sixteen As-As single bonds (Fig. 2a), (5) eight As-Pd single bonds (Fig. 2b), and (6) one global σ bond (Fig. 2c). These assignments are beautifully borne out from the AdNDP analyses, which describe 92 out of 94 electrons as As or Pd lone-pairs (Fig. 3a–c) and As-As and As-Pd 2c-2e σ bonds (Fig. 3d–e), and leave the remaining two electrons completely delocalized (Fig. 3f).

As for the nature of the HOMO (Fig. 2c), on each PdAs_4_ unit it is completely delocalized and completely bonding, due to radial overlaps of As p and Pd d/s/p AOs; see Table 2. The Pd and As components in the HOMO are roughly 55% versus 45%, indicating highly covalent Pd-As bonding. In effect, the HOMO may be cut in halves so that each PdAs_4_ unit has a delocalized 5c-1e σ bond. The shape of such 5c-1e σ bond (half bond, to be exact) is consistent with σ-aromaticity, although its electron-counting is only half of that from the (4*n* + 2) Hückel rule, with *n* = 0. Effectively, a 5c-1e σ half bond within a PdAs_4_ unit in **1** can be considered to be 50% as aromatic as a 5c-2e σ bond. Thus, cluster **1** possesses σ aromaticity. We stress that this conclusion is reached entirely on the bases of the CMO and AdNDP analyses.

**Table 2:** Composition analysis for selected canonical molecular orbitals (CMOs) in *D*_2*h*_ [Pd_2_As_14_]^4−^ (**1**) and [Au_2_Sb_14_]^4−^ (**2**) clusters at the PBE0/def2-TZVP level.

Complex	CMO^a^	Pd/Au (%)			As/Sb (%)	
		s	p	d	s	p
[Pd_2_As_14_]^4−^	HOMO	7.84	7.22	39.1	1.44	43.48
[Au_2_Sb_14_]^4−^	HOMO	—	1.66	—	—	87.52
	HOMO-4	—	1.86	1.68	2.16	92.76
	HOMO-10	—	—	—	7.12	91.12
	HOMO-12	—	3.54	3.86	—	89.76
	HOMO-13	23.78	2.86	16.32	9.0	47.1
	HOMO-25	31.82	—	6.02	—	45.0

^a^These are the only delocalized CMOs in clusters **1** and **2**.

NICS calculations are indeed in line with the idea of σ-aromaticity in **1** (Table S2), whose NICS(1) and NICS(1)_zz_ values are −23.31 and −16.43 ppm, respectively, at 1 Å above the PdAs_4_ fragment. It is known that electron density affects the result of NICS analysis. We thus also calculated the corresponding NICS(1) and NICS(1)_zz_ values for the model neutral complex **3**, which are −49.85 and −43.46 ppm, respectively, at 1 Å above the PdAs_4_ fragment. The NICS values for both tetraanion **1** and neutral **3** clusters are highly negative, consistent with σ-aromaticity. It is emphasized here that the CMO analyses and electron counting are the most fundamental tools in elucidating aromaticity of a molecular system; complementary analyses (such as NICS calculations) only offer additional or independent support for the assessment. Since NICS as a criterion of aromaticity has been documented to fail in a number of cases (in particular in metal clusters), we are inclined to state that the correlation between the NICS values and the assessment of aromaticity in this case can be “*a mere coincidence*”^52^, as one referee suggests. Indeed, we are not claiming σ aromaticity in this system because it has negative NICS values; rather σ aromaticity is established through extensive CMO and AdNDP analyses, as stated above.

Apart from NICS, we also performed calculations using the quantum theory of atoms-in-molecules (QTAIM)^53^. Here, the Multiwfn^54^ program is used to generate the para-delocalization index (PDI^55^; As3-As7-As9-As12-As14-As16) and the multicenter bond aromaticity index (MCI)^56^. The PDI and MCI data for tetraanion **1** and model neutral cluster **3** are presented in Table S2, which are compared with those of [Au_2_Sb_14_]^4−^ (**2**) and benzene (C_6_H_6_). It is shown that: (a) PDI_π_ and MCI_π_ of species **1**–**3** are zero, consistent with the nature that these species do not have π aromaticity; (b) The PDI_σ_ and MCI_σ_ values are 0.030–0.121 and 0.271–0.315, respectively, consistent with σ aromaticity. Note that these PDI_σ_/MCI_σ_ values are to be compared to the PDI_π_ (0.093) and MCI_π_ (0.383) of benzene, because species **1**–**3** are σ aromatic and benzene has π aromaticity.

In summary, excluding the 44 lone-pairs (or nonbonding electrons), tetraanion **1** is a 50-electron system. Of these, the 2c-2e σ bonds consume 48. Notably, the Pd and As(iii) centers participate evenly in Pd-As 2c-2e σ bonds, with the Pd_2_ dumbbell contributing 8 electrons and the eight As(iii) centers collectively matching the remaining 8. The only delocalized CMO uses the remaining 2 electrons, which are equally split in between two PdAs_4_ units, rendering σ-aromaticity for **1**. Our analyses indicate that no Pd-Pd covalent bond is present in **1**, in contrast to the claim in ref. 24.

Owing to the fact that the Pd_2_ dumbbell in **1** has six Pd 4d based nonbonding CMOs or lone-pairs (Fig. S5a) and that each Pd center carries one extra charge, the oxidation state can be formally viewed as Pd(III), as proposed by Eichhorn and coworkers. However, this is only an oversimplified ionic picture, in which cluster **1** is described as two [As_7_]^5−^ cages linked via a Pd_2_^6+^ dumbbell^24^. The actual bonding in **1** deviates fundamentally from the above picture and indeed the eight Pd-As bonds appear to be highly covalent, with half versus half contributions from Pd_2_ and As_8_ in the cube (Fig. 2b), which is consistent with their closeness in electronegativity (Pd: 2.20; As: 2.18). In the updated point-of-view, we have two [As_7_]^−^ cages interconnected by Pd_2_^2−^, where Pd is in formal oxidation state of Pd(−I).

### [Au_2_Sb_14_]^4−^ Cluster as a σ-Aromatic Model “Charged Molecular Alloy”

**Figure 4:**
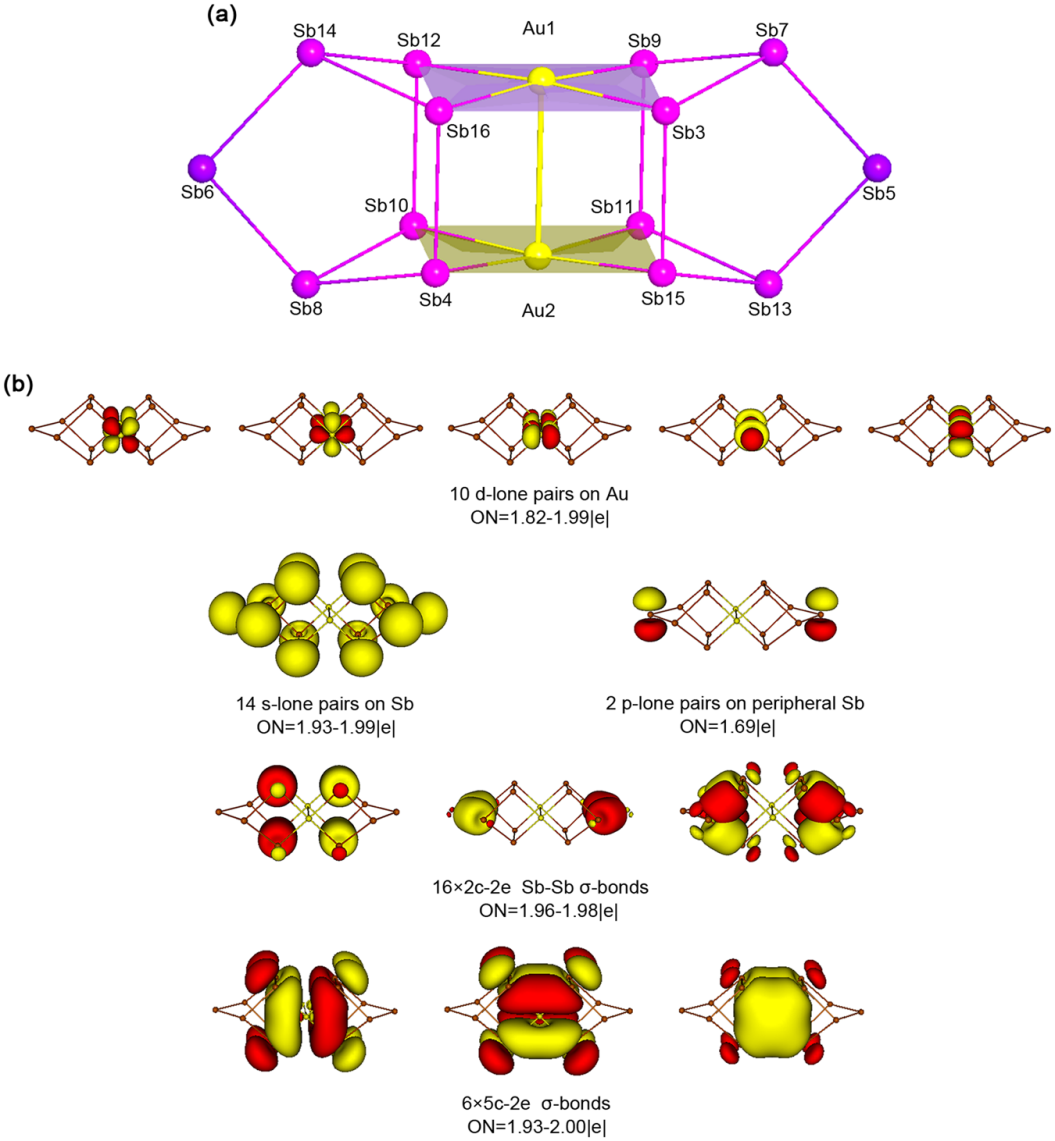
Model cluster [Au_2_Sb_14_]^4−^ (**2**). (**a**) Optimized *D*_2*h*_ structure at the PBE0/def2-TZVP level. (**b**) Bonding pattern on the basis of AdNDP analysis. Occupation numbers (ONs) are shown.

The intriguing bonding and σ-aromaticity in *D*_2*h*_ [Pd_2_As_14_]^4−^ (**1**) have stimulated us to “design” additional charged molecular alloys. One example is the *D*_2*h*_ [Au_2_Sb_14_]^4−^ (**2**) cluster, which differs from **1** by two electrons (Au 5d^10^6s^1^ versus Pd 4d^10^). Structure-wise, the *D*_2*h*_ structure of [Au_2_Sb_14_]^4−^ is a true minimum on the potential energy surface and can be considered as a model cluster (Fig. 4a). However, the bonding of **1** and **2** differs markedly.

*D*_2*h*_ [Au_2_Sb_14_]^4−^ (**2**) has 96 valence electrons. Excluding the Au 5d^10^, Sb 5s^2^, two bridging Sb 5p lone-pairs, which use 52 electrons (Figs 4b and S6), cluster **2** can be viewed as a 44-electron system. Of these, the Sb-Sb 2c-2e σ single bonds consume 32 electrons, whose corresponding CMOs are shown in Fig. S7a and AdNDP elements in Fig. 4b. Not surprisingly, these sixteen CMOs show one-to-one correspondence to those of **1** (Fig. S7a versus Fig. 2a). The extra charges in **2** are distributed on the two bridging Sb centers and, to a lesser extent, two Au centers (Table S3), which are similar to **1** except that the charges around Au seems to be more evenly distributed over the AuSb_4_ units. In fact, the HOMO and HOMO-1 of **2** are based on Sb(iii) and Sb(i) centers, suggesting that the net charge on Au is probably due to intramolecular charge transfer (rather than the extra charges), which is in line with the difference in electronegativity between Au (2.54) and Sb (2.05). The peripheral Sb-Sb links within AuSb_4_ are elongated (Sb12-Sb16: 3.76 Å; Sb16-Sb3: 3.94 Å) with respect to Sb-Sb single bonds, and thus there is no direct Sb-Sb covalent bonding in the periphery. Furthermore, no classical 2c-2e Au-Sb single bonds are present in **2** and the Au-Au interaction is minimal, the latter showing a “magic” distance of 3.11 Å, which is typical for aurophilicity^50, 51^ and in line with the calculated Wiberg index (0.186; Table S3). In contrast to **1**, cluster **2** has 12 delocalized electrons.

Consistent with the bonding elements discussed above, the calculated bond distances of Sb-Sb and Au-Sb are 2.77–2.88 and 2.73 Å, respectively, which are compared to their recommended values^48^ of single bonds: 2.80 and 2.64 Å. Clearly, Sb-Sb single bonds are well defined, whereas Au-Sb bond is elongated with respect to single bond, indicating a delocalized bonding system in the AuSb_4_ units.

**Figure 5:**
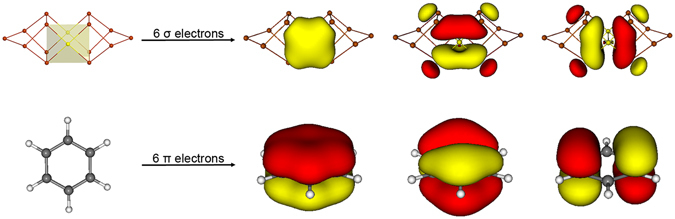
Comparison of one of the σ sextets in [Au_2_Sb_14_]^4−^ (**2**) and the prototypical π sextet in benzene. These σ or π bonds are generated from the adaptive natural density partitioning (AdNDP) analysis; two sets of σ sextet are present in [Au_2_Sb_14_]^4−^ (**2**).

The 12 delocalized electrons in cluster **2** occupy six CMOs as depicted in Fig. S7b. Their corresponding AdNDP elements are shown in Fig. 4b, bottom row. Thus, each square-planar AuSb_4_ unit in **2** supports a completely bonding 5c-2e σ bond, as well a pair of partially bonding ones. The three σ bonds define a σ sextet and follow the (4*n* + 2) electron-counting of the Hückel rule, which is closely analogous to the π sextet in benzene; see comparisons in Fig. 5. It is thus imperative to claim σ-aromaticity for *D*_2*h*_ [Au_2_Sb_14_]^4−^ (**2**), with *n* = 1. Indeed, the calculated NICS values for **2** are negative (Table S2).

We believe σ-aromaticity is governing the stability of *D*_2*h*_ [Au_2_Sb_14_]^4−^ (**2**), because otherwise there would be neither Sb-Sb nor Au-Sb bonding within the two AuSb_4_ fragments. We have recently described a synthetic [Au_2_Sb_16_]^4−^ compound^38^, which is highly distorted with quasi-*C*_2_ point group symmetry, also featuring two σ sextets. In carefully designed and controlled syntheses, we anticipate the *D*_2*h*_ σ-aromatic [Au_2_Sb_14_]^4−^ (**2**) complex to be made as bulk materials in laboratory.

Lastly, we briefly comment on the oxidation state of Au in **2**. Here, the two-pairs of extra charges occupy the Sb(i) and Sb(iii) sites; see HOMO-1 and HOMO (Figs S6 and S7). The Au center does not form 2c-2e bonds with Sb, nor does it bound covalently with the other Au center. Thus, the Au centers in **2** should be considered as Au(0) and cluster **2** is best described as two [Sb_7_]^2−^ linked via Au_2_^0^. While [E_7_]^3−^ (E = P, As, Sb)^21^ are known as naked cluster anions, the [As_7_]^−^ or [Sb_7_]^2−^ cages in **1** and **2** do not appear odd. Our recent work^36, 37^ shows that all-metal aromatic [Sb_3_Au_3_Sb_3_]^3−^ sandwich complex has two [Sb_3_]^1.5−^ ligands, although [Sb_3_]^3−^ is routine in solution syntheses.

## Concluding Remarks

In conclusion, we have presented a density-functional theory study on the structures and chemical bonding of a synthetic “charged molecular alloy”, *D*_2*h*_ [Pd_2_As_14_]^4−^ (**1**), as well as those of a *D*_2*h*_ [Au_2_Sb_14_]^4−^ (**2**) model cluster. Both **1** and **2** possess two distorted square-planar MX_4_ units that are virtually parallel to each other, and a dumbbell M_2_ interconnects two X_7_ cages to form the charged molecular alloys. While **1** and **2** differ only for two valence electrons, their nature of bonding differs markedly. There are only two delocalized σ electrons in cluster **1**, conforming to the electron-counting for σ-aromaticity. In contrast, cluster **2** possesses two delocalized σ sextets (each being situated on an AuSb_4_ unit), which render σ-aromaticity for **2**. No covalent Pd-Pd or Au-Au bonding is observed in **1** and **2**. The Pd and Au centers in **1** and **2** clusters are virtually isolated, single atoms in nature^50, 51^, with specific oxidation states, which make them interesting model systems for the so-called “single atom catalysis”^57^. The designer σ-aromatic *D*_2*h*_ [Au_2_Sb_14_]^4−^ (**2**) cluster also invites forth-coming synthetic effort.

### Methods Section

Cluster structure of [Pd_2_As_14_]^4−^ (**1**) is optimized using density-functional theory (DFT)^40^ at the PBE0/def2-TZVP level^39, 58^, whose performance has been tested recently in relevant compound systems^36, 37^. We also constructed and optimized the structure of [Au_2_Sb_14_]^4−^ (**2**) at the same level of theory. To elucidate chemical bonding in the systems, the CMO and AdNDP^41^ analyses were performed and the results were visualized using the Molekel program^59^. NBO analysis^42^ was carried out to obtain the natural atomic charges and Wiberg bond indices. NICS^43^ calculations were performed to assess the nature of aromaticity in the systems. The AdNDP analyses were performed using the AdNDP program^41^ and all other calculations and analyses were carried out using the Gaussian 09 software package^60^. Orbital composition analysis was performed using the Multiwfn program^54^.

## Electronic supplementary material

suppl info
