# Supplementary material for: Chemical Bonding and σ-Aromaticity in Charged Molecular Alloys: [Pd2As14]4− and [Au2Sb14]4− Clusters
Source: Sci Rep. 2017 Apr 11;7:791. doi: 10.1038/s41598-017-00867-5 (PMC5429782; doi:10.1038/s41598-017-00867-5)
Supplement: Supplementary file 1 — suppl info [file 41598_2017_867_MOESM1_ESM.pdf]

SUPPLEMENTARY INFORMATION

Chemical Bonding and σ-Aromaticity in Charged Molecular Alloys: $[Pd_2As_{14}]^{4-}$ and $[Au_2Sb_{14}]^{4-}$ Clusters

Xue-Rui You,[†] Lin-Yan Feng,[†] Rui Li,[†] and Hua-Jin Zhai*[,†,‡]

[†]*Nanocluster Laboratory, Institute of Molecular Science, Shanxi University, Taiyuan 030006, China*

[‡]*State Key Laboratory of Quantum Optics and Quantum Optics Devices,*

Shanxi University, Taiyuan 030006, China

*E-mail: hj.zhai@sxu.edu.cn

Table S1. Cartesian coordinates for D_{2h} $[Pd_2As_{14}]^{4-}$ (**1**) and D_{2h} $[Au_2Sb_{14}]^{4-}$ (**2**) at the PBE0/def2-TZVP level.

Table S2. Calculated nucleus independent chemical shift (NICS) values (in ppm), as well as other electron delocalization and aromaticity indices (PDI and MCI), for $[Pd_2As_{14}]^{4-}$ (**1**), $[Au_2Sb_{14}]^{4-}$ (**2**), $[Pd_2As_{14}K_4]$ (**3**), and benzene (C_6H_6) at the PBE0/def2-TZVP level.

Table S3. Optimized geometric structure of model $[Au_2Sb_{14}]^{4-}$ (**2**) cluster at PBE0/def2-TZVP level. Also presented are Wiberg bond indices and natural atomic charges via the natural bond orbital (NBO) analysis.

Figure S1. Optimized structures of (a) D_{2h} $[Pd_2As_{14}]^{4-}$ (**1**) and (b) a model neutral cluster C_i $[Pd_2As_{14}K_4]$ (**3**) at the PBE0/def2-TZVP level, along with all of their bond distances (in Å). Cluster **3** is based on cluster **1**, with four K^+ counter-ions being attached around the two Pd centers and two As(i) bridging atoms of tetraanion **1**.

Cluster **3** is closely relevant to and yet much simpler than the synthetic complex $[K([2.2.2]crypt)]_4[Pd_2As_{14}]$ 5en.

Figure S2. View of $[K([2.2.2]crypt)]_4[Pd_2As_{14}]$ 5en down the *a* axis, based on ref. 24. Bond distances (in Å) are shown for comparison with the calculated results in Figures S1 and S3.

Figure S3. Optimized structure of D_{2h} $[Pd_2As_{14}]^{4-}$ (**1**) cluster using the C-PCM in calculations at PBE0/def2-TZVP level, which takes into account of the solvation effects (ethylendiamine). Bond distances (in Å) and natural charges (in red color) are shown, which are consistent with the results that neglect the solvent effects (Fig. S1a; Table 1).

Figure S4. Energy level diagrams of the bare $[Pd_2As_{14}]^{4-}$ (**1**, D_{2h}) tetraanion with solvation effects (left) and the model $[Pd_2As_{14}K_4]$ (**3**, C_i) neutral cluster (right) at the PBE0/def2-TZVP level.

Figure S5. Pictures of canonical molecular orbitals (CMOs) of $[Pd_2As_{14}]^{4-}$ (**1**) associated with (a) nonbonding Pd 4d CMOs or lone-pairs, (b) fourteen As $4s^2$ lone-pairs, and (c) two bridging As 4p lone-pairs. These CMOs are calculated at the PBE0/def2-TZVP level, consuming 44 electrons out of 94 in total in the system.

Figure S6. Pictures of canonical molecular orbitals (CMOs) of $[Au_2Sb_{14}]^{4-}$ (**2**) associated with (a) ten Au $5d^{10}$ lone-pairs, (b) fourteen Sb $5s^2$ lone-pairs, and (c) two bridging Sb 5p lone-pairs. These CMOs are calculated at the PBE0/def2-TZVP level, consuming 52 electrons out of 96 in total in the system.

Figure S7. Pictures of all CMOs in $[Au_2Sb_{14}]^{4-}$ (**2**) calculated at the PBE0/def2-TZVP level, with the exclusion of the lone-pairs in Figure S6. These CMOs suggest that **2** is a 44-electron system, consisting of (a) sixteen two-center two-electron (2c-2e) Sb−Sb σ bonds and (b) six delocalized σ CMOs on the two $AuSb_4$ fragments.

Table S2. Calculated nucleus independent chemical shift (NICS) values (in ppm), as well as other electron delocalization and aromaticity indices (PDI and MCI), for $[Pd_2As_{14}]^{4-}$ (**1**), $[Au_2Sb_{14}]^{4-}$ (**2**), $[Pd_2As_{14}K_4]$ (**3**), and benzene (C_6H_6) at the PBE0/def2-TZVP level. [a]

	$[Pd_2As_{14}]^{4-}$	$[Pd_2As_{14}K_4]$	$[Au_2Sb_{14}]^{4-}$	C_6H_6
NICS(1)	**–23.31**	**–49.85**	**–7.05**	**–22.43**
NICS(1)zz	**–16.43**	**–43.46**	**–6.90**	**–49.13**
PDI	0.030	0.030	0.121	0.107
PDI_σ	**0.030**	**0.030**	**0.121**	0.014
PDI_π	0	0	0	**0.093**
MCI	0.315	0.271	0.247	0.637
MCI_σ	**0.315**	**0.271**	**0.247**	0.255
MCI_π	0	0	0	**0.383**

[a] Numbers in **boldface** are those that should be compared between $[Pd_2As_{14}]^{4-}$ (**1**) / $[Au_2Sb_{14}]^{4-}$ (**2**) / $[Pd_2As_{14}K_4]$ (**3**) and benzene, because the first three species are σ aromatic only, where benzene has π aromaticity.

Table S3. Optimized geometric structure of model $[Au_2Sb_{14}]^{4-}$ (**2**) cluster at PBE0/def2-TZVP level. Bond distances are in Å. Also presented are Wiberg bond indices and natural atomic charges (in |e|) via the natural bond orbital (NBO) analysis.

	Au(1)–Au(2)	Au(1/2)–Sb(iii)[a]	Sb(i)–Sb(ii)[a]	Sb(ii)–Sb(iii)[a]	Sb(iii)–Sb(iii)[a,b]
Bond distance	3.11	2.73	2.77	2.85	2.88
Wiberg index	0.186	0.602	1.061	0.912	1.030
Natural charge[c]	Au		Sb		
			Sb(i)	Sb(ii)	Sb(iii)
	−0.417		−0.629	−0.188	−0.144

[a] The Sb atoms in $[Au_2Sb_{14}]^{4-}$ (**2**) are classified into three types. Sb(i): two bridging As atoms; Sb(ii): four Sb atoms that are tricoordinated with Sb; Sb(iii): eight Sb atoms linked to Au.

[b] Only four Sb(iii)–Sb(iii) bonds are present in **2**, which interconnect two square-planar $AuSb_4$ units; See Figure 4(a).

[c] Charge per Au or Sb atom.

Figure S1. Optimized structures of (a) D_{2h} $[Pd_2As_{14}]^{4-}$ (**1**) and (b) a model neutral cluster C_i $[Pd_2As_{14}K_4]$ (**3**) at the PBE0/def2-TZVP level, along with all of their bond distances (in Å). Cluster **3** is based on cluster **1**, with four K^+ counter-ions being attached around the two Pd centers and two As(i) bridging atoms of tetraanion **1**. Cluster **3** is closely relevant to and yet much simpler than the synthetic complex $[K([2.2.2]crypt)]_4[Pd_2As_{14}]$ ·5en.

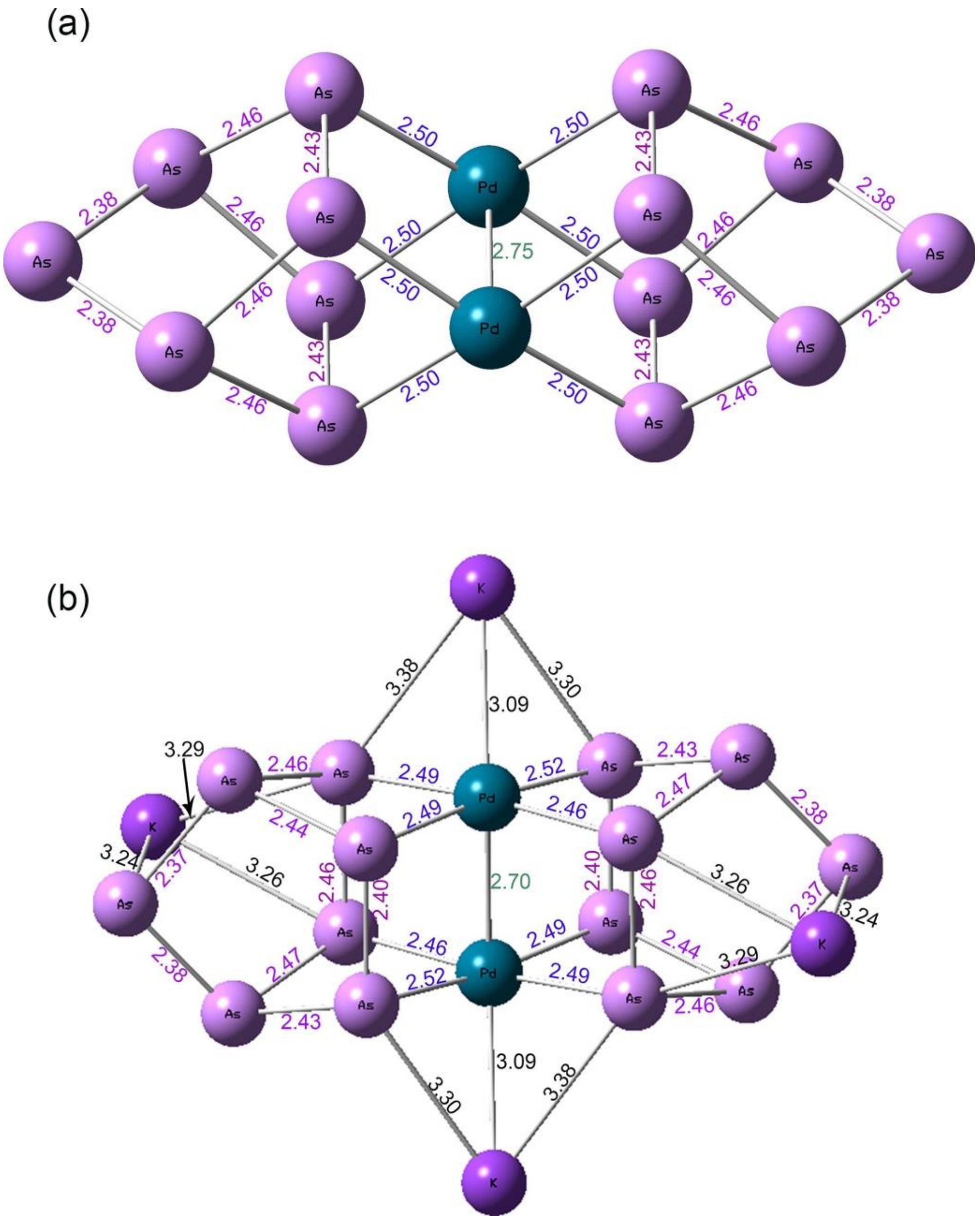

Figure S2. View of $[K([2.2.2]crypt)]_4[Pd_2As_{14}]$ 5en down the *a* axis, based on ref. 24. Bond distances (in Å) are shown for comparison with the calculated results in Figures S1 and S3.

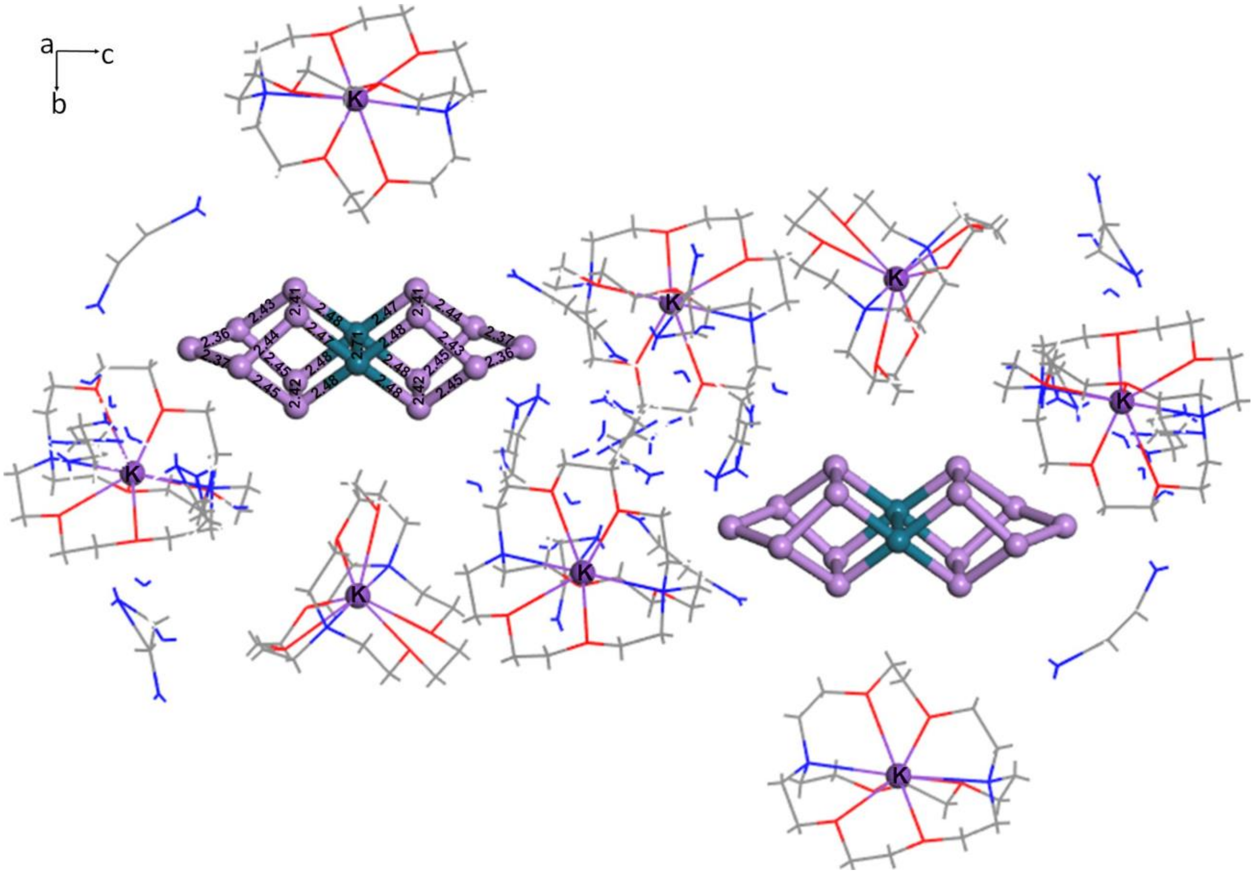

Figure S3. Optimized structure of D_{2h} $[Pd_2As_{14}]^{4-}$ (**1**) cluster using the C-PCM in calculations at PBE0/def2-TZVP level, which takes into account of the solvation effects (ethylendiamine). Bond distances (in Å) and natural charges (in red color) are shown, which are consistent with the results that neglect the solvent effects (Fig. S1a; Table 1).

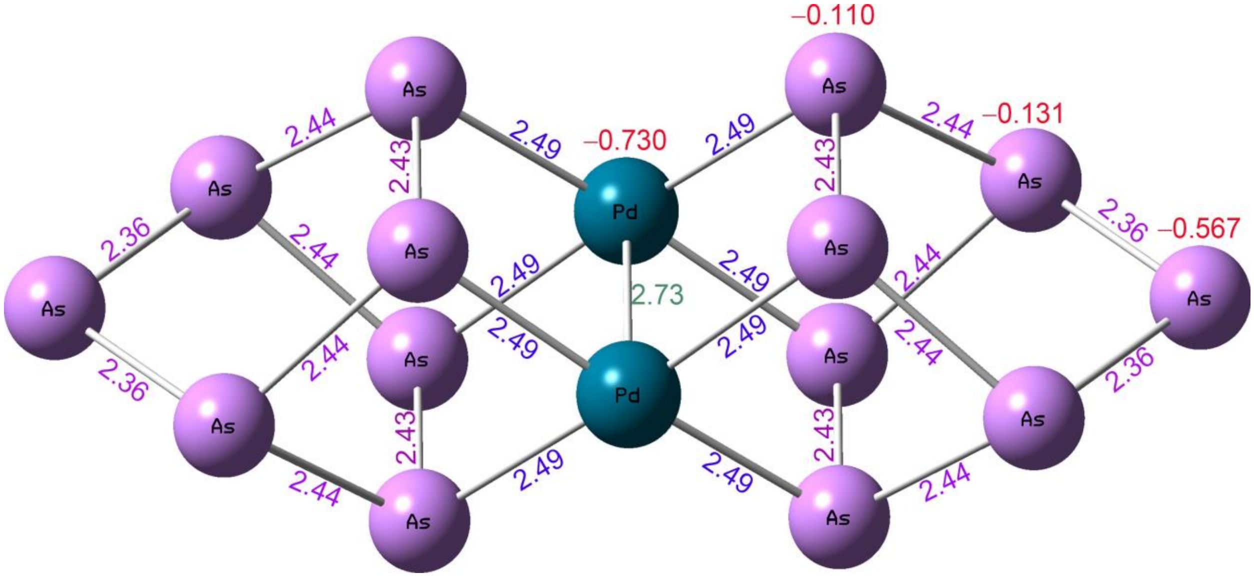

Figure S4. Energy level diagrams of the bare $[Pd_2As_{14}]^{4-}$ (**1**, D_{2h}) tetraanion with solvation effects (left) and the model $[Pd_2As_{14}K_4]$ (**3**, C_i) neutral cluster (right) at the PBE0/def2-TZVP level. HOMO−1 and HOMO−2 in **1** are very close in energy. The same set of orbitals are present in the two systems, except that their energy orders vary slightly.

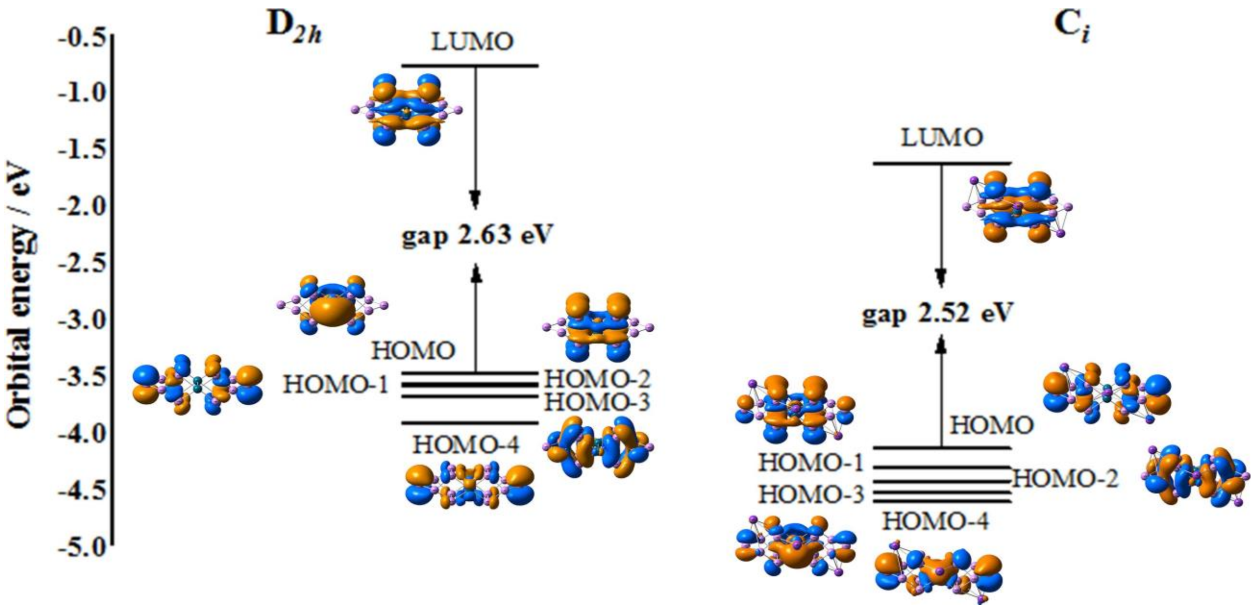

Figure S5. Pictures of canonical molecular orbitals (CMOs) of $[Pd_2As_{14}]^{4-}$ (**1**) associated with (a) nonbonding Pd 4d CMOs or lone-pairs, (b) fourteen As $4s^2$ lone-pairs, and (c) two bridging As 4p lone-pairs. These CMOs are calculated at the PBE0/def2-TZVP level, consuming 44 electrons out of 94 in total in the system.

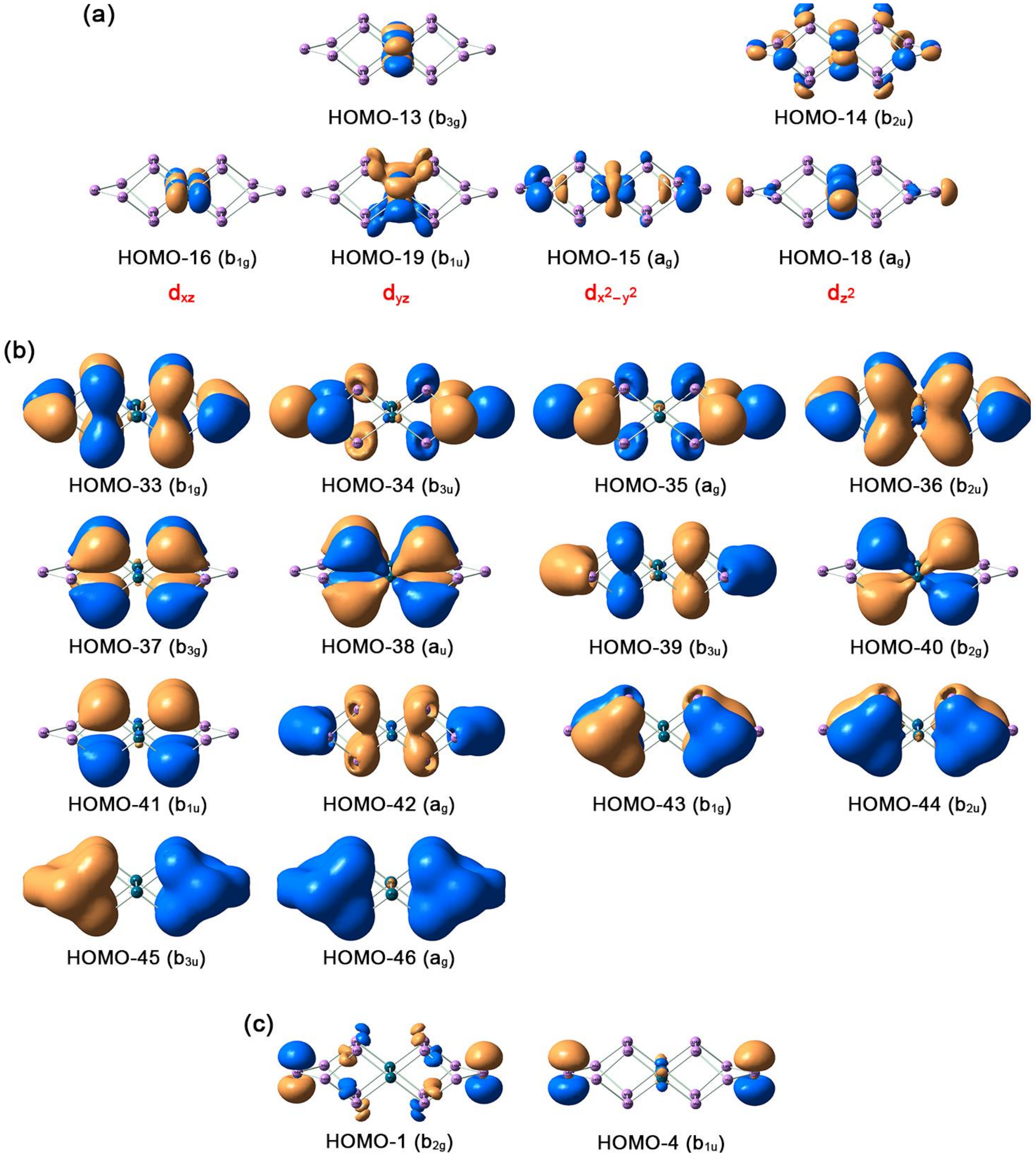

Figure S6. Pictures of canonical molecular orbitals (CMOs) of $[Au_2Sb_{14}]^{4-}$ (**2**) associated with (a) ten Au $5d^{10}$ lone-pairs, (b) fourteen Sb $5s^2$ lone-pairs, and (c) two bridging Sb 5p lone-pairs. These CMOs are calculated at the PBE0/def2-TZVP level, consuming 52 electrons out of 96 in total in the system.

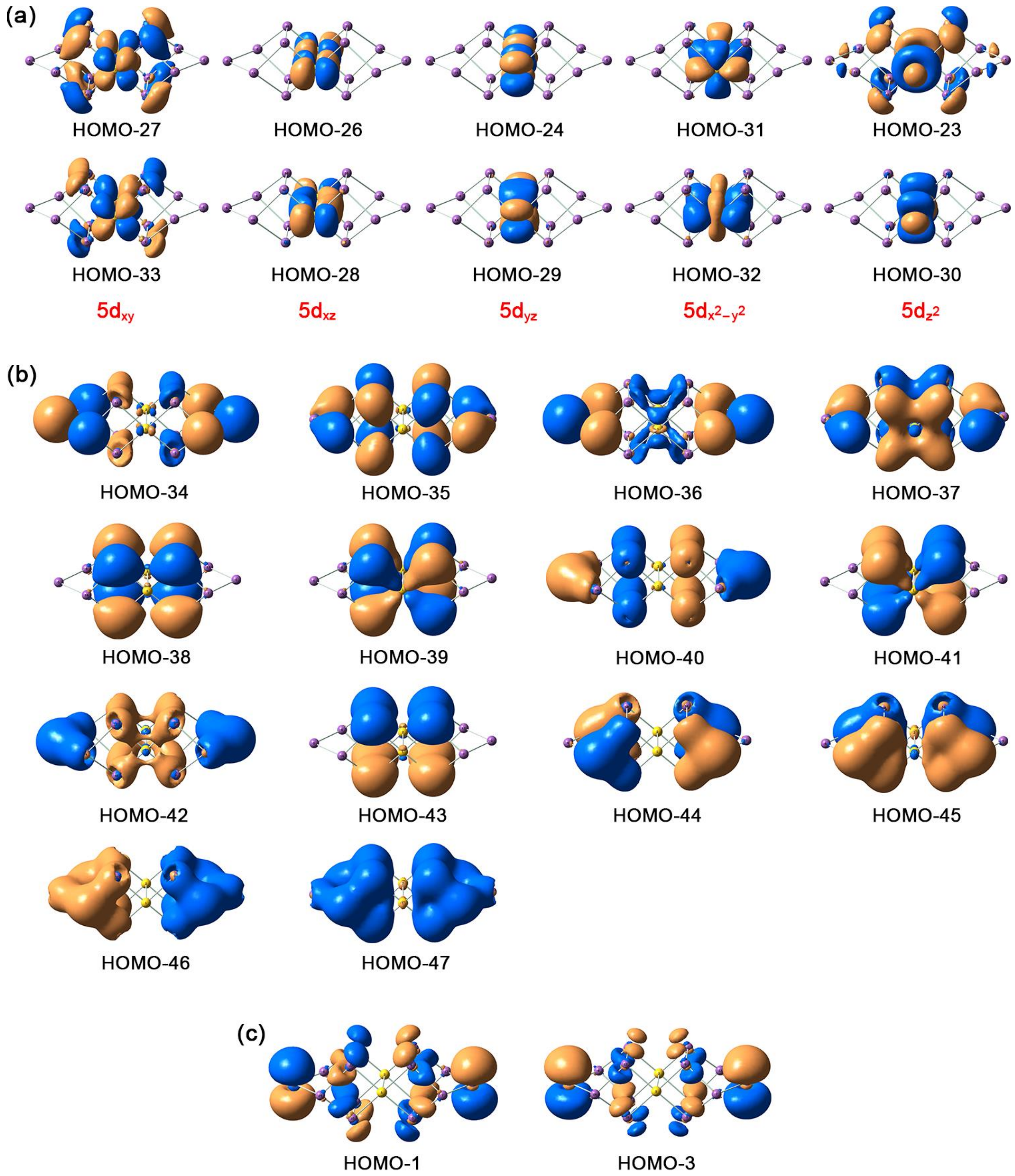

Figure S7. Pictures of all CMOs in $[Au_2Sb_{14}]^{4-}$ (**2**) calculated at the PBE0/def2-TZVP level, with the exclusion of the lone-pairs in Figure S6. These CMOs suggest that **2** is a 44-electron system, consisting of (a) sixteen two-center two-electron (2c-2e) Sb–Sb σ bonds and (b) six delocalized σ CMOs on the two $AuSb_4$ fragments.

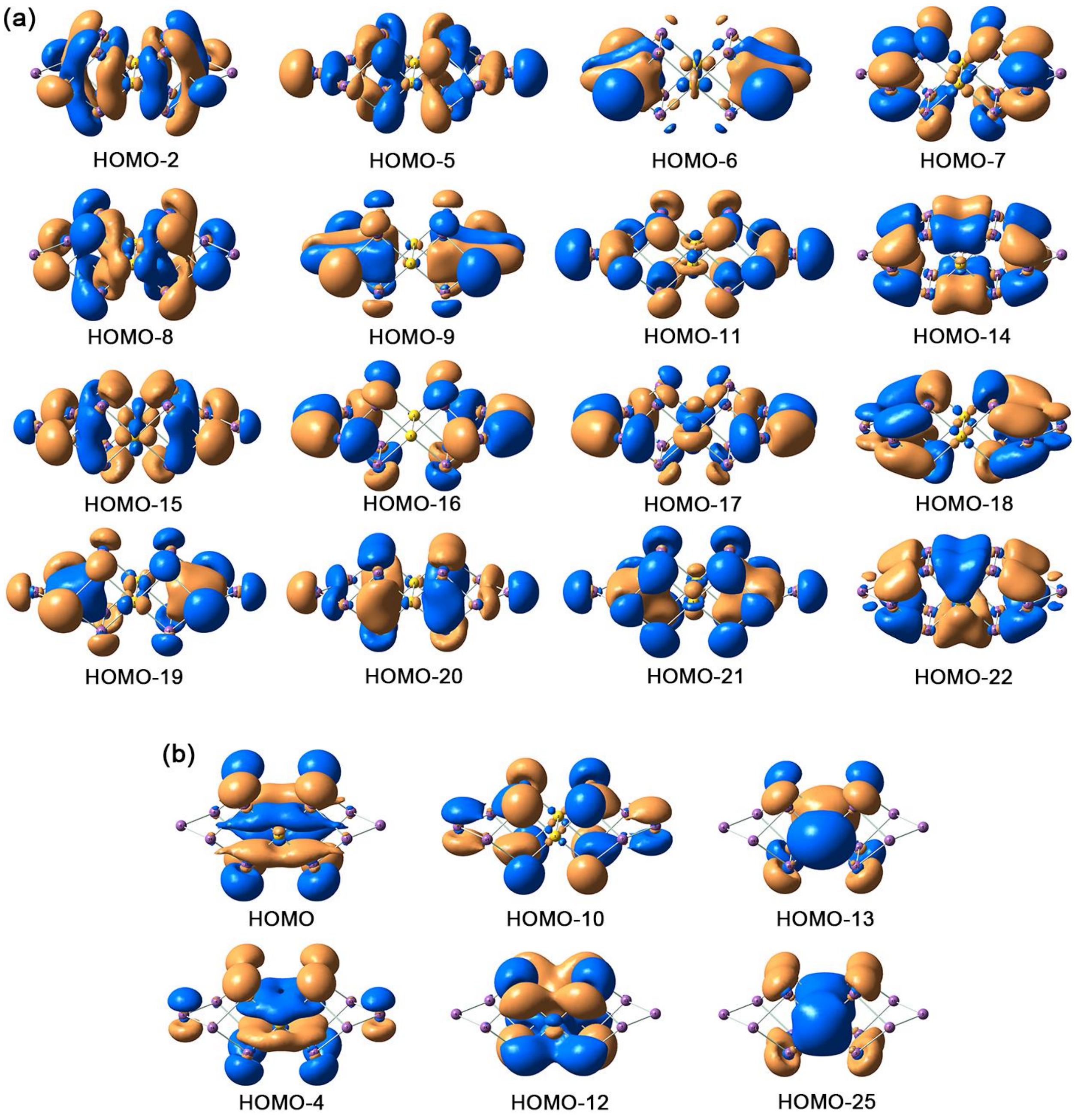

Table S1. Cartesian coordinates (in Å) for D_{2h} $[Pd_2As_{14}]^{4-}$ (**1**) and D_{2h} $[Au_2Sb_{14}]^{4-}$ (**2**) at the PBE0/def2-TZVP level.

(1) D_{2h} $[Pd_2As_{14}]^{4-}$

Pd	0.00000000	1.37612200	0.00000000
Pd	0.00000000	-1.37612200	0.00000000
As	1.96258100	1.21622100	1.54664000
As	-1.96258100	-1.21622100	1.54664000
As	5.35629600	0.00000000	0.00000000
As	-5.35629600	0.00000000	0.00000000
As	3.78581400	1.78556400	0.00000000
As	-3.78581400	-1.78556400	0.00000000
As	1.96258100	1.21622100	-1.54664000
As	-1.96258100	-1.21622100	-1.54664000
As	1.96258100	-1.21622100	-1.54664000
As	-1.96258100	1.21622100	-1.54664000
As	3.78581400	-1.78556400	0.00000000
As	-3.78581400	1.78556400	0.00000000
As	1.96258100	-1.21622100	1.54664000
As	-1.96258100	1.21622100	1.54664000

(2) D_{2h} $[Au_2Sb_{14}]^{4-}$

Au	-0.00000000	1.55519900	-0.00000000
Au	0.00000000	-1.55519900	-0.00000000
Sb	1.97168793	1.44135654	1.88090391
Sb	-1.97168793	-1.44135654	1.88090391

Sb	5.86568903	-0.00000000	-0.00000000
Sb	-5.86568903	-0.00000000	-0.00000000
Sb	4.01853372	2.06010226	0.00000000
Sb	-4.01853372	-2.06010226	0.00000000
Sb	1.97168793	1.44135654	-1.88090391
Sb	-1.97168793	-1.44135654	-1.88090391
Sb	1.97168793	-1.44135654	-1.88090391
Sb	-1.97168793	1.44135654	-1.88090391
Sb	4.01853372	-2.06010226	0.00000000
Sb	-4.01853372	2.06010226	0.00000000
